# Retraction: Prenatal Cocaine Exposure Increases Synaptic Localization of a Neuronal RasGEF, GRASP-1 via Hyperphosphorylation of AMPAR Anchoring Protein, GRIP

**DOI:** 10.1371/journal.pone.0266630

**Published:** 2022-03-30

**Authors:** The PLOS ONE Editors

Following the publication of this article [1], concerns were raised regarding results presented in Figures 1, 2, 4, and 6. Specifically,

- There appear to be horizontal and vertical irregularities suggestive of splice lines in the following panels:
  - Figure 1c, within lane 4 of the Caspase 3 panel.
  - Figure 2a, just above the 90kDa marker of the GRASP-1 panel.
  - Figure 2a, around each individual band in the GRIP1 panel.
  - Figure 6a, between lanes 7–8 of the GluR2 panel.
- Further irregularities have been detected in the background of the following panels:
  - Figure 4a Rap1 panel, near the lower right edge of the panel there appears to be a truncated fragment of a double band.
  - Figure 6a GRASP-1 panel, when levels are adjusted to visualize background, the density of the background noise directly surrounding the bands in lanes 5 and 6 does not appear to match the background noise density elsewhere in the blot.
  - Figure 6a GRIP1 panel, when levels are adjusted to visualize background, there appear to be sharp horizontal and vertical discontinuities in lane 1.

The corresponding author disagreed with the above concerns. They stated that each panel was obtained from a single blot and that the observations are likely the result of image compression artifacts or experimental artifacts such as gel or reagent remnants, or patches intrinsic to the nitrocellulose membranes.

The corresponding author provided image data to support their published western blot results in this [1] and other *PLOS ONE* articles [2–5]. Per PLOS’ assessment of the data files, the pixel patterns in background areas of blot images provided for multiple panels in [1–5] appear more similar than would be expected for data obtained in independent experiments. The corresponding author stated that the repetitive features in the background noise of the underlying data are likely the result of scanner artifacts.

The data and comments provided to PLOS did not resolve the concerns about the integrity and reliability of the reported data. In light of these issues, the *PLOS ONE* Editors retract this article.

RGN and HYW did not agree with the retraction. KB, MK, and EF either did not respond directly or could not be reached. HYW stands by the article’s findings.
